# Proteomic study of *Akkermansia muciniphila* and *Bifidobacterium* species co-culture under different carbon sources

**DOI:** 10.3389/fmicb.2025.1666747

**Published:** 2025-11-06

**Authors:** Jordy Evan Sulaiman, Yuewei Zhan, Shuchen Wang, Ka Lun Lai, James Ho Wa Li, Daniel Ye Yutong, Karl Wah Keung Tsim, Kenneth King Yip Cheng, Yong Lai, Henry Lam

**Affiliations:** 1 Department of Health Technology and Informatics, The Hong Kong Polytechnic University, Kowloon, Hong Kong SAR, China; 2 Research Institute for Future Food (RiFood), The Hong Kong Polytechnic University, Kowloon, Hong Kong SAR, China; 3 Department of Chemical and Biological Engineering, The Hong Kong University of Science and Technology, Clear Water Bay, Hong Kong, Hong Kong SAR, China; 4 Center for Chinese Medicine, Division of Life Science, The Hong Kong University of Science and Technology, Clear Water Bay, Hong Kong, Hong Kong SAR, China; 5 State Key Laboratory of Molecular Neuroscience, Division of Life Science, The Hong Kong University of Science and Technology, Clear Water Bay, Hong Kong, Hong Kong SAR, China

**Keywords:** *Akkermansia muciniphila*, *Bifidobacterium*, carbon sources, communities, inter-species interaction, glycan, proteomics, co-culture

## Abstract

*Akkermansia muciniphila* and *Bifidobacterium* spp. are major probiotic strains that have been shown to improve host metabolism and treat metabolic diseases. Previous studies have proposed formulating these probiotics as a therapeutic product to improve efficacy, but how they affect the growth and protein expression of each other in response to different nutrient environments remains unexplored. Here, we performed label-free quantitative proteomics on *A. muciniphila* and two *Bifidobacterium* species, *Bifidobacterium longum* and *Bifidobacterium breve*, in the presence of different carbon sources. *Akkermansia muciniphila* displayed distinct growth profiles when co-cultured with *B. breve* and *B. longum* in media containing monosaccharides (glucose and N-acetyl-D-glucosamine) or mucin. *Akkermansia muciniphila* led to reduced abundance of *B. longum* in co-culture compared to monoculture, irrespective of whether the media contained monosaccharides or mucin. By contrast, *B. breve* led to reduced abundance of *A. muciniphila* in co-culture compared to monoculture in the presence of the monosaccharides but not in the mucin medium. Proteomics analysis revealed that *B. breve* induced substantial alterations in the protein expression of *A. muciniphila* when cultured in the media with monosaccharides, but the two species minimally affected each other’s protein expression when cultured in the mucin medium. By screening health-relevant dietary fibers, we discovered that arabinoxylan selectively boosts the growth of *B. longum* in monoculture and co-culture. Notably, in the presence of arabinoxylan, *B. longum* promotes the growth of *A. muciniphila* and increases the expression of Amuc_1100 protein, leading to the enhancement of barrier integrity of intestinal epithelial cells. In sum, we demonstrated that glycans shape the growth and proteome profiles of *A. muciniphila* and *B. breve* or *B. longum* co-cultures and highlight that dietary fibers can be utilized to improve the functionality of the probiotic community.

## Introduction

In recent years, people have realized the importance of gut homeostasis and intestinal immunity in promoting metabolic health and protecting against diseases. Supplementation of beneficial bacteria as probiotics or live biotherapeutic products (LBPs) has gained massive interest due to their potential in modulating the gut microbiome and intestinal functions, which subsequently confer multiple beneficial effects to distal organs (O’Toole et al., 2017). For instance, *Akkermansia muciniphila* (AM) is a mucin-degrading species that maintains gut health and influences the balance of the gut microbiota (Derrien et al., 2004). The abundance of AM is high in the gut of healthy individuals (Hagi and Belzer, 2021), but decreased in people with obesity and type 2 diabetes (Liu et al., 2017; Dao et al., 2016; Yassour et al., 2016; Brunkwall and Orho-Melander, 2017). AM has been highlighted as a potential candidate for ameliorating metabolic diseases (Niu et al., 2024; Yan et al., 2021). Its outer membrane protein Amuc_1100 binds to Toll-like receptor-2 (TLR-2) in the intestinal epithelial cells (IECs), and the downstream signaling pathway leads to the improvement of the epithelial tight junction and triggers anti- and pro-inflammatory cytokines that prevent obesity, insulin resistance, and inflammation in the visceral adipose tissue (Si et al., 2022). In addition, the glucagon-like protein P9 secreted by AM binds to the intercellular adhesion molecule 2 on the surface of L cells, inducing the secretion of glucagon-like peptide-1 (GLP-1) that regulates energy balance by triggering insulin release from the pancreas and improving glucose homeostasis. Although AM supplementation has been shown to exert positive effects in rodents (Everard et al., 2013; Wu et al., 2025) and humans (Depommier et al., 2019; Zhang et al., 2025) with metabolic diseases, its efficacy is still not optimal. In some cases, supplementation of live AM is less effective than its pasteurized form (Depommier et al., 2019) and fails to modulate gut microbiota composition (Everard et al., 2013; Depommier et al., 2019; Zhang et al., 2025). These suggest that combining AM with specific diets or other bacterial species that promote the abundance and colonization of AM in the gut could improve its efficacy and ability to modulate the dysbiotic gut microbiome.

Besides AM, *Bifidobacterium* species, including *B. lactis*, *B. bifidum*, *B. adolescentis*, *B. breve* (BB), and *B. longum* (BL), have been used as probiotics due to their positive effects on the metabolic health and gut immunity of obese individuals (Solito et al., 2021; Minami et al., 2015; Schellekens et al., 2021; Chichlowski et al., 2020; Fukuda et al., 2011). Combining AM with *Bifidobacterium* spp. could improve efficacy through potential positive interactions, increased health benefits by targeting different regulatory pathways, and enhanced robustness with respect to environmental context. The combination of AM and *Bifidobacterium* spp. has been proposed as a food supplement (Vedor et al., 2023) or for the treatment of metabolic diseases (Nian et al., 2023). However, the nature and mechanism of their interactions remain largely unexplored. Previous studies inferred the correlations between AM and *Bifidobacterium* spp. using 16S rRNA sequencing or metagenomic sequencing data from stool samples (Hagi and Belzer, 2021; Wang et al., 2011; Shang et al., 2018; Alard et al., 2016; Toscano et al., 2017). However, these analyses are phenomenological rather than theoretical and hence have limited predictive capability. Besides, it is also unable to separate the observed effects from host factors, environmental factors, and the other interacting gut commensals. *In vitro* co-culture experiments could decipher how the presence of a species affects the growth and protein regulation of another species under diverse conditions (Sulaiman et al., 2024; Sulaiman et al., 2025; Ostrem Loss et al., 2023; Feng et al., 2022). This could yield insights into the interactions that positively impact the growth of AM and *Bifidobacterium* spp. and the production of their beneficial proteins or metabolites.

Glycan utilization is a major driver of gut microbiota interactions. While negative interactions can stem from competition for specific glycans (Tuncil et al., 2017), utilization of glycan breakdown products (i.e., the liberated monosaccharides) can enhance the growth of some community members (Rakoff-Nahoum et al., 2014; Rakoff-Nahoum et al., 2016). Since AM and *Bifidobacterium* spp. possess numerous glycan-degrading enzymes (Bell and Juge, 2021), different glycans would affect their interactions in communities and might be utilized to manipulate their abundances for therapeutic applications. How different glycans affect biological processes beyond the expression of glycan utilization enzymes, and how the growth of AM and *Bifidobacterium* spp. is different in co-culture vs. monoculture in the presence of those glycans, are still unclear. Proteomics is a suitable tool to investigate this problem and to obtain a global view of the cellular processes. It allows us to directly measure the expression of the glycan utilization enzymes, along with other affected proteins and biological processes.

Here, we investigated the growth and proteome profiles of AM and two *Bifidobacterium* spp., BB and BL, in the presence of different carbon sources. AM led to reduced growth of BL in co-culture compared to monoculture, both in the presence of monosaccharides (glucose and N-acetyl-D-glucosamine) and mucin. By contrast, BB led to reduced growth of AM in co-culture compared to monoculture only in the presence of monosaccharides, but not mucin. Proteomics analysis showed that BB led to substantial alterations in the proteome of AM when cultured in media with monosaccharides, but they did not affect one another when cultured in mucin medium. By screening through health-relevant dietary fibers, we identified that arabinoxylan (AX) can selectively boost the growth of BL in monoculture and co-culture. Notably, in the presence of AX, BL promoted the growth of AM and increased the expression of the beneficial protein Amuc_1100, leading to the enhancement of barrier integrity of IECs. Overall, our study revealed that glycans modulate the growth and proteome profiles of AM and BB or BL co-cultures and demonstrated the ability of AX to enhance the ability of the AM + BL pairwise community in improving the barrier integrity of IECs.

## Results

### *Akkermansia muciniphila* displays distinct growth profiles with *B. breve* and *B. longum* in the presence of mucin compared to monosaccharides

**Figure 1:**
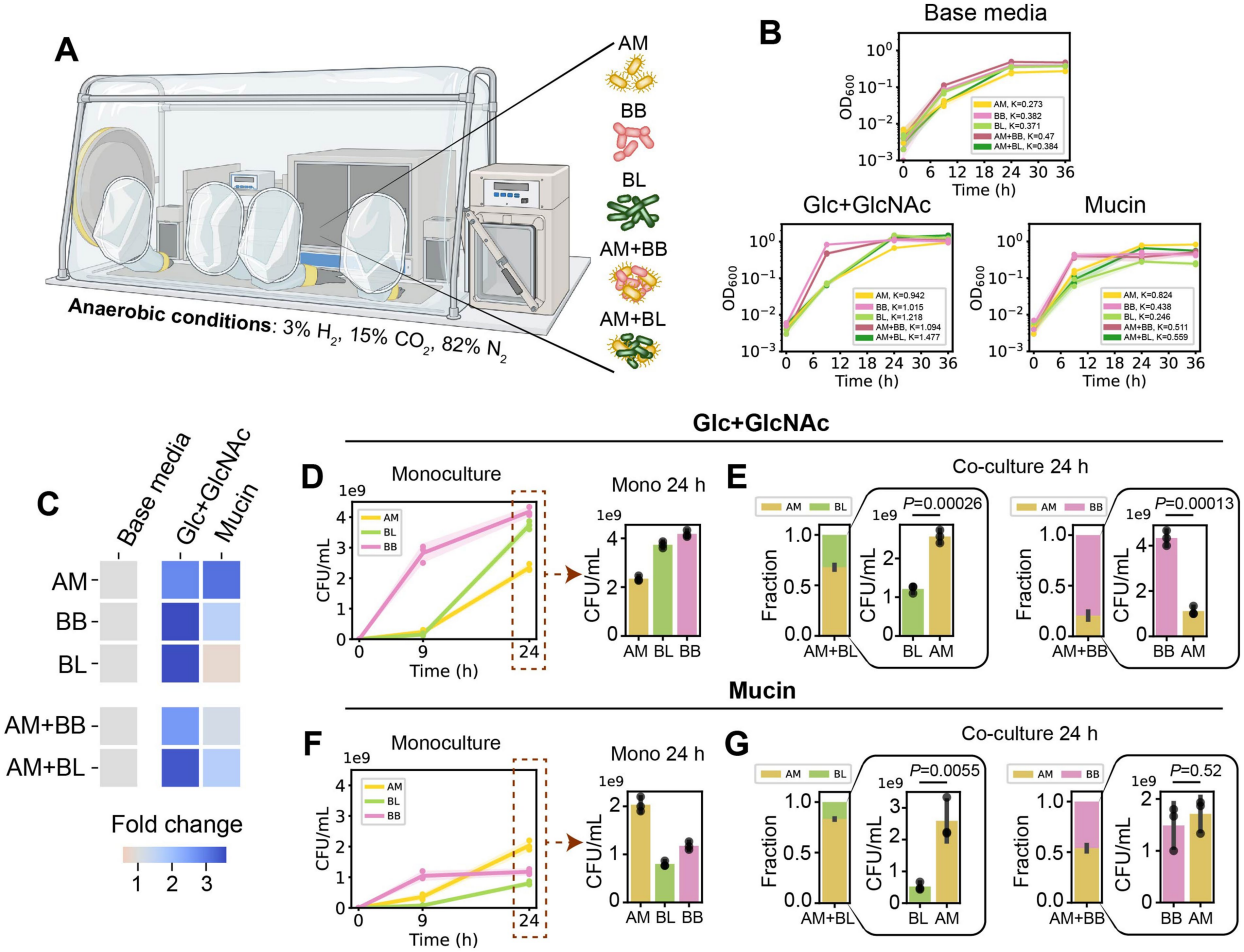
*Akkermansia muciniphila* exhibits distinct growth profiles in co-culture with *B. breve* and *B. longum* in Glc + GlcNAc or mucin media. **(A)** *A. muciniphila* (AM), *B. breve* (BB), and *B. longum* (BL) were cultured in monocultures or pairwise co-cultures (AM + BB or AM + BL) in an anaerobic chamber (see Methods). **(B)** OD_600_ of AM, BB, BL, AM + BB, AM + BL cultured in base media (BHI), base media supplemented with glucose (Glc) and N-acetyl-D-glucosamine (GlcNAc), and base media supplemented with porcine gastric mucin, measured over 36 h. Individual data points were shown. Lines represent the mean, and shading represents standard deviation (s.d.). The maximum carrying capacity (K) of each species in the respective media is shown in the figure panels. **(C)** Heatmap of fold change of the Area Under the Curve (AUC) for each monoculture and co-culture when cultured in the presence of Glc + GlcNAc or mucin compared to growth in the base media alone. AUC data were extracted from panel **(B)** and Supplementary Figure S1B. **(D)** Absolute abundance of AM, BB, and BL monocultures in base media supplemented with Glc + GlcNAc as measured by CFU counting (*n* = 3). **(E)** Stacked bar plot of relative abundance and bar plot of absolute abundance of AM, BB, and BL in AM + BB or AM + BL co-cultures after 24 h of growth in the base media supplemented with Glc + GlcNAc as measured by CFU counting. Each bar represents the average relative or absolute abundance of each species, and the error bars represent s.d. (*n* = 3). *p*-values from a two-sided unpaired Student *t*-test of absolute abundance between AM and BL or BB are shown. **(F)** Absolute abundance of AM, BB, and BL monocultures after 24 h of growth in base media supplemented with mucin as measured by CFU counting (*n* = 3). **(G)** Stacked bar plot of relative abundance and bar plot of absolute abundance of AM, BB, and BL in AM + BB or AM + BL co-cultures after 24 h of growth in the base media supplemented with mucin as measured by CFU counting. Each bar represents the average relative or absolute abundance of each species, and the error bars represent s.d. (*n* = 3). *p*-values from a two-sided unpaired Student *t*-test of absolute abundance between AM and BL or BB are shown.

To understand how glycans influence the growth of probiotic strains in monocultures and co-cultures, we characterized three bacterial species: AM BAA-835, BB JCM1192, and BL JCM1217. We chose brain-heart infusion (BHI) as the base media for our system since it can minimally support the growth of all species (allowing us to obtain sufficient samples for proteomics analysis), and their growth could be further enhanced by supplementing other carbon sources (Supplementary Figure S1A). We characterized their growth anaerobically in the base media containing either glucose and N-acetyl-D-glucosamine (Glc + GlcNAc) or mucin in monocultures and co-cultures (Figure 1A). Mucin is one of the host-derived glycans in the gut, serving as a continuous endogenous source of microbiota-accessible carbohydrates for resident microbes. While AM can utilize mucin as its sole carbon and nitrogen source (Kim et al., 2022), *Bifidobacterium* spp. have varying degrees of mucin degradation capabilities (Ruas-Madiedo et al., 2008; Abe et al., 2010). It is unclear how mucin affects the growth of AM in co-culture with *Bifidobacterium* spp. and how it impacts other cellular processes beyond the expression of mucin utilization-related enzymes.

Based on the growth profiles, all species reached maximum population size within 24 h (i.e., entered the stationary phase) (Figures 1B,C; Supplementary Figure S1B). By comparing the growth in the presence and absence of Glc + GlcNAc or mucin, we observed that all species can utilize Glc + GlcNAc. By contrast, mucin can be utilized by AM, but not by BL. While BB can also utilize mucin, the amount of growth was much lower than that of AM. Growth characterization via optical density (OD_600_) measurements or CFU counting resulted in a consistent trend across all media conditions (Figures 1D,F; Supplementary Figure S1C). While OD_600_ can be used to assess the total growth of bacteria in communities, CFU counting using selective plates enables growth measurement of individual species within the communities. In co-culture, AM displayed differences in growth profile with the two *Bifidobacterium* spp. (Figures 1E,G). After 24 h of growth, the relative abundance of AM in AM + BB co-culture was low in the presence of Glc + GlcNAc (~20%) and increased in the presence of mucin (~54%). The absolute abundance of AM (quantified by CFU counting) was lower in AM + BB co-culture compared to AM monoculture in the Glc + GlcNAc media (Figures 1D,E), whereas the abundance of AM was similar in AM + BB co-culture and AM monoculture in the mucin media (Figures 1F,G). By contrast, AM dominated BL in the AM + BL co-culture in the presence of both carbon sources after 24 h of growth (relative abundance of ~68 and 83% in Glc + GlcNAc and mucin, respectively). In both media, the absolute abundance of BL was lower in AM + BL co-culture compared to the BL monoculture (Figures 1D–G).

### *Bifidobacterium breve* alters the proteome of *A. muciniphila* when supplemented with monosaccharides but not mucin

To investigate proteome alterations of AM, BB, and BL in co-culture vs. monoculture across the two carbon sources and how they are associated with changes in the growth profiles, we subjected them to proteomic analysis (See Methods, Supplementary Figures S2, S3). AM, BB, BL, AM + BB, and AM + BL were cultured for 24 h in media containing different carbon sources since this duration allows all species to reach maximum population size (Figure 1B), followed by protein extraction and LC–MS/MS analysis.

**Figure 2:**
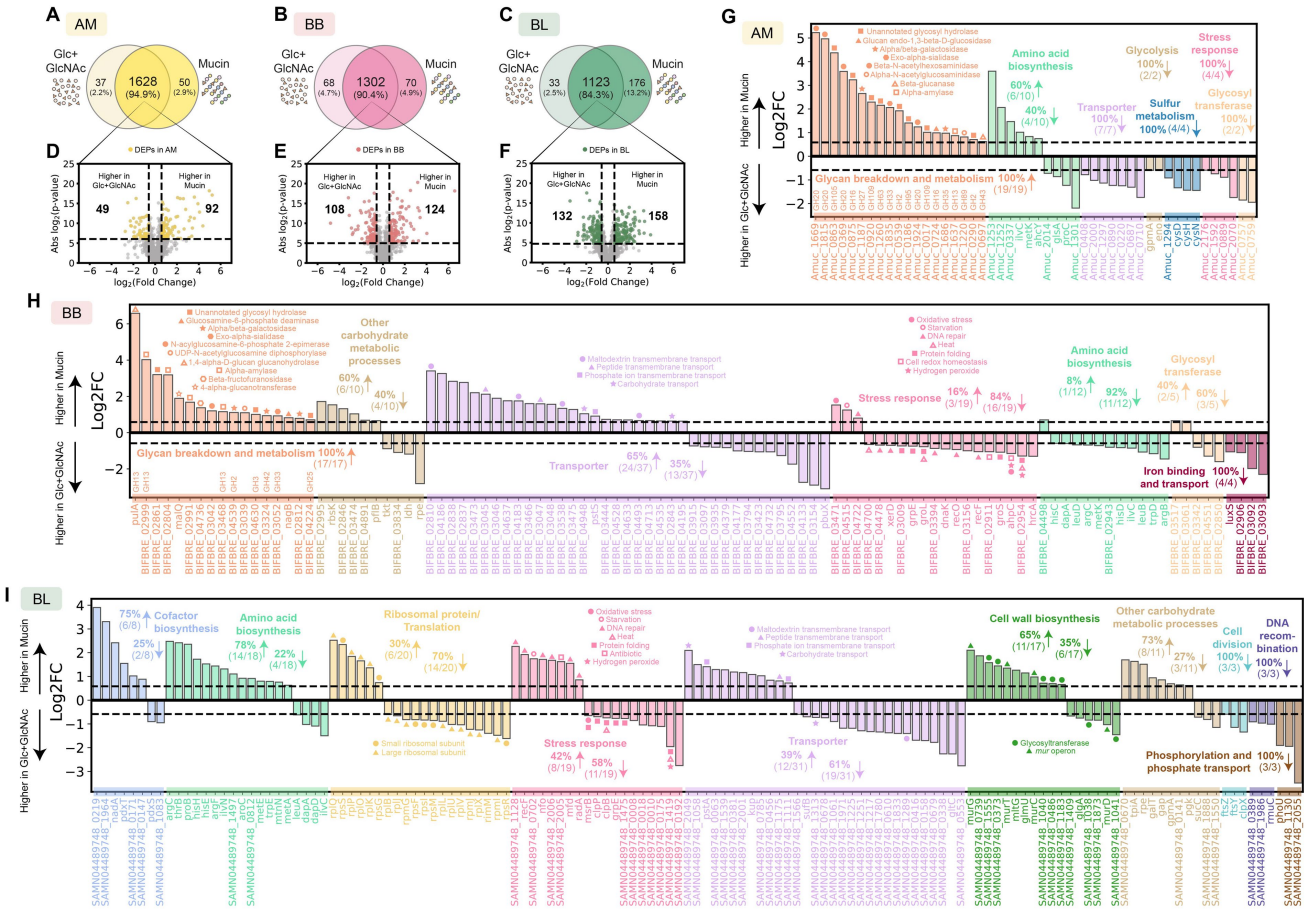
Proteome alterations of *A. muciniphila, B. breve*, and *B. longum* in the presence of mucin compared to glucose and GlcNAc. **(A–C)** Venn diagram for proteome comparison of AM **(A)**, BB **(B)**, and BL **(C)** in the presence of mucin vs. Glc + GlcNAc. Proteins shown are detected in two out of three biological replicates. **(D-F)** Volcano plot for AM **(D)**, BB **(E)**, and BL **(F)** in the presence of mucin vs. Glc + GlcNAc. Benjamini–Hochberg (BH) multiple testing correction was applied to the *p*-values from a two-sided unpaired Student *t*-test to control the false discovery rate (FDR) at 10%. In addition to the BH-corrected *p*-value constraint, differentially expressed proteins (DEPs) are defined to be those with an absolute fold change greater than 1.5, corresponding to the colored dots. The list of DEPs for AM, BB, and BL is shown in Supplementary Tables S2, S3, and S4, respectively. **(G–I)** Log-transformed fold changes of selected DEPs that were expressed higher in mucin (positive values) or Glc + GlcNAc (negative values). Vertical dashed lines indicate a 1.5-fold change.

In monoculture, AM exhibited higher expression of many glycosyl hydrolases required for mucin degradation, such as GH20 (Xu et al., 2020; Chen et al., 2019), GH35 (Kosciow and Deppenmeier, 2019; Guo et al., 2018), and GH16 (Crouch et al., 2020) enzyme families in the presence of mucin compared to Glc + GlcNAc (Figures 2A,D,G; Supplementary Table S2). Other glycosyl hydrolases that were expressed higher by AM in the presence of mucin (e.g., GH2, GH13, GH33, GH43, GH89, GH95) have also been shown to be important for growth in mucin, as their deletion led to profound growth defects in mucin medium (Davey et al., 2023). In the presence of Glc + GlcNAc, AM displayed higher expression of transporter proteins and those that play a role in glycolysis, sulfur metabolism, and stress response. BB also exhibited higher expression of proteins involved in glycan breakdown and metabolism in the presence of mucin than Glc + GlcNAc (Figures 2B,E,H; Supplementary Table S3). By contrast, the expression of proteins for stress response, amino acid biosynthesis, and iron binding and transport was higher in the presence of Glc + GlcNAc. Since BL cannot utilize mucin, it did not show differential expression of enzymes for glycan breakdown in the presence of mucin compared to Glc + GlcNAc (Supplementary Table S4). The number of differentially expressed proteins (DEPs) of BL in mucin vs. Glc + GlcNAc was higher compared to AM or BB (Figures 2C,F,I). The DEPs for BL in mucin vs. Glc + GlcNAc are involved in cofactor biosynthesis, amino acid biosynthesis, translation, stress response, cell wall biosynthesis, cell division, DNA recombination, metabolic processes, and transporters.

**Figure 3:**
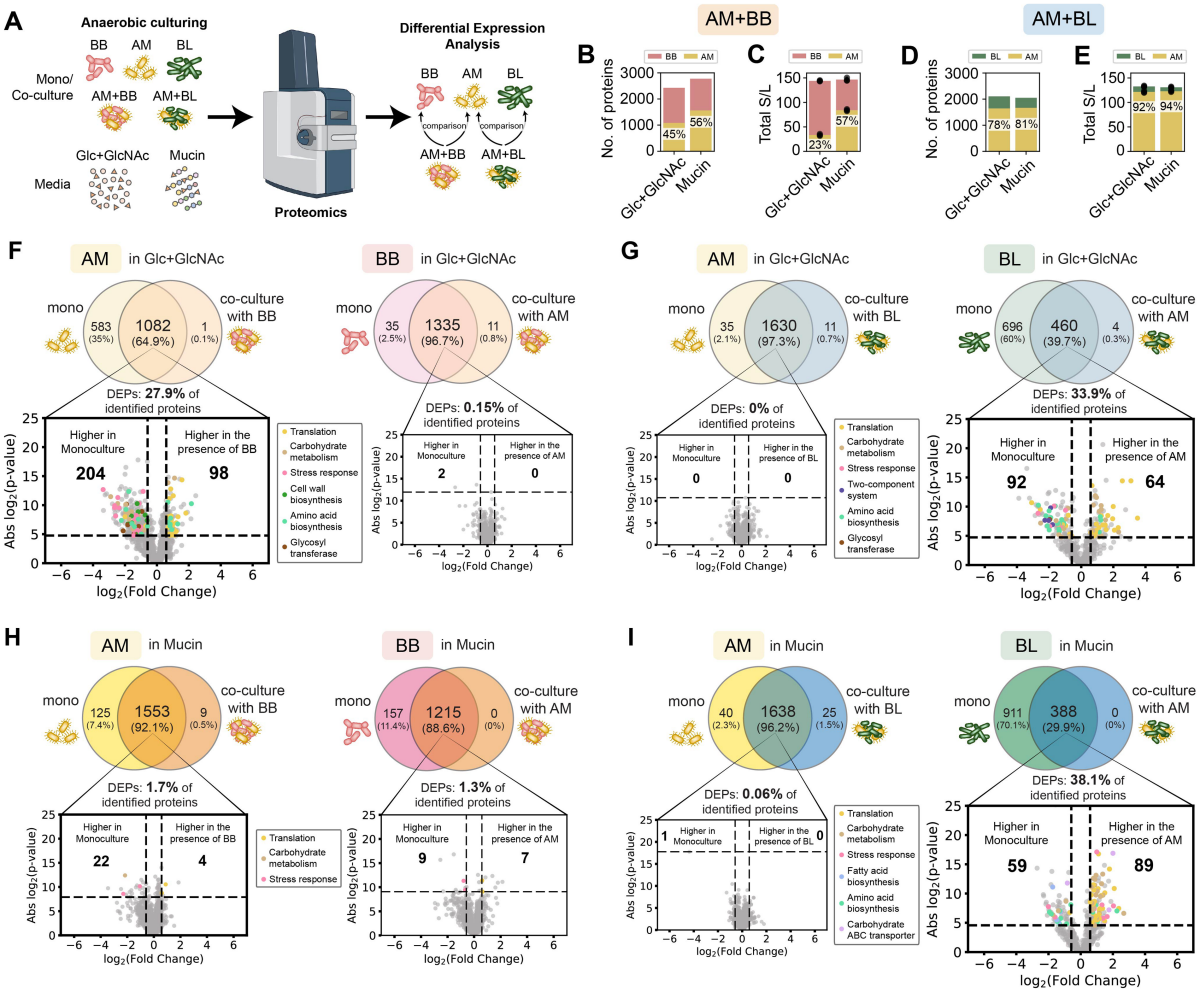
Proteome profiling of *A. muciniphila* in co-culture with *B. breve* or *B. longum* vs monoculture in the Glc + GlcNAc or mucin media. **(A)** Schematic of the proteomics experiment workflow. **(B–E)** Number of proteins **(B)** and total normalized spectral counts of the whole proteins **(C)** in the AM + BB co-culture, and the number of proteins **(D)** and total normalized spectral counts of the whole proteins **(E)** in the AM + BL co-culture in the presence of different carbon sources. The percentage shows the fraction of AM’s protein in the co-culture or the fraction of total normalized spectral counts of proteins belonging to AM in the co-culture. **(F–I)** Proteome comparison of AM or BB in monoculture vs. AM + BB co-culture in Glc + GlcNAc **(F)**, AM or BL in monoculture vs. AM + BL co-culture in Glc + GlcNAc **(G)**, AM or BB in monoculture vs. AM + BB co-culture in mucin **(H)**, and AM or BL in monoculture vs. AM + BL co-culture in mucin **(I)**. Proteins shown in the Venn diagrams are detected in two out of three biological replicates. The volcano plot shows proteins that are expressed higher in co-culture vs. monoculture, and proteins belonging to different biological processes are shown with different colors. Benjamini–Hochberg (BH) multiple testing correction was applied to the *p*-values from a two-sided unpaired Student *t*-test to control the false discovery rate (FDR) at 10%. In addition to the BH-corrected *p*-value constraint (horizontal dashed line), differentially expressed proteins (DEPs) are defined to be those with an absolute fold change greater than 1.5 (vertical dashed lines). The list of DEPs is shown in Supplementary Tables S5–S11.

When analyzing proteomics data of AM + BB and AM + BL co-culture (Figure 3A), there is a possibility that a peptide sequence belongs to the proteins of both AM and BB or BL (i.e., shared peptides). To evaluate the severity of this issue, we performed *in silico* tryptic digestion of the whole proteome of AM, BB, and BL using the Uniprot database and generated a list of all possible shared peptides between AM and BB or AM and BL (see Methods). The percentage of theoretical shared peptides across the proteome for AM + BB, AM + BL, and BB + BL is 0.222% (779/351,301), 0.218% (774/354,827), and 14.8% (46,083/310,822), respectively. This is consistent with the fact that AM is not closely related to BB and BL, whereas BB and BL are closely related. Most of these shared peptides between AM and BB or BL are short (~5 amino acids), which was the threshold we set in our database searching (Supplementary Figure S4). We then searched whether the peptides identified in the co-culture proteomic samples contain any of the shared peptides. There is a very low number of shared peptides detected across our proteomic datasets, ranging from 13 to 19 in each sample. To take a conservative approach, we assigned the spectra of the shared peptide to both species in the co-culture. Unconfident peptide assignment with a low number of sibling peptides will be removed by the Trans-Proteomics Pipeline (TPP) algorithm.

For the proteomics analysis, we first compared the proteome profile of the samples under different carbon sources to observe how resource environments impact protein expression. Then, we compared the proteome profile of each species in monoculture versus co-culture under the same carbon source to reveal the effect of the partner species on the protein expression of a particular strain. For co-culture samples, the expression of a protein was normalized with the expression of the whole proteins of the species that express that particular protein. In AM + BB co-culture, AM has higher protein identification in the presence of mucin (56%, 1,562 out of 2,777 total proteins) compared to Glc + GlcNAc (45%, 1,083 out of 2,429 total proteins) (Figure 3B). Similarly, the total protein expression of AM is also higher in mucin than Glc + GlcNAc when co-cultured with BB (57% vs. 23% of the total co-culture protein expression) (Figure 3C). By contrast, the protein identification and total protein expression of AM are similarly high when co-cultured with BL in Glc + GlcNAc and mucin (Figures 3D,E). The number of proteins identified for AM when co-cultured with BL in Glc + GlcNAc and mucin are 1,641 out of 2,105 (78%) and 1,663 out of 2,051 (81%) respectively, whereas the total protein expression of AM when co-cultured with BL in Glc + GlcNAc and mucin are 92 and 94% of the total co-culture protein expression, respectively. These results are consistent with the higher relative abundance of AM in co-culture with BB when cultured in mucin than in GlcNAc, and the higher fraction of AM in AM + BL co-culture both in the presence of Glc + GlcNAc and mucin (Figures 1D–G).

In the presence of Glc + GlcNAc, AM did not significantly affect the proteome of BB, whereas BB led to substantial alterations in the proteome of AM (~28% DEPs, Figure 3F; Supplementary Figure S5; Supplementary Tables S5, S6, S12, S13). Many of the up-regulated proteins were those involved in translation, amino acid biosynthesis, and carbohydrate metabolism, whereas the down-regulated proteins were glycosyl transferases and those that play a role in amino acid biosynthesis, carbohydrate metabolism, stress response, and cell wall biosynthesis. By contrast, BB and AM did not significantly alter the protein expression of each other in the presence of mucin (Figure 3H; Supplementary Tables S7, S8). In the presence of Glc + GlcNAc, BL did not significantly affect the proteome of AM, whereas AM led to substantial alterations in the proteome of BL (~34% DEPs) (Figure 3G; Supplementary Table S9). Similarly, BL did not affect the proteome of AM, and AM substantially altered the proteome of BL in the presence of mucin (~38% DEPs) (Figure 3I; Supplementary Tables S10, S11).

In sum, the two nutrient environments (Glc + GlcNAc and mucin media) affect the proteome expression of AM and BB in co-culture, but not AM and BL. While BB induced substantial alterations of AM’s proteome in Glc + GlcNAc medium, this was not observed in the mucin medium. On the other hand, AM induced proteome alterations in BL in both Glc + GlcNAc and mucin media.

### *Bifidobacterium longum* displays increased fitness in co-culture with *A. muciniphila* in the presence of arabinoxylan

**Figure 4:**
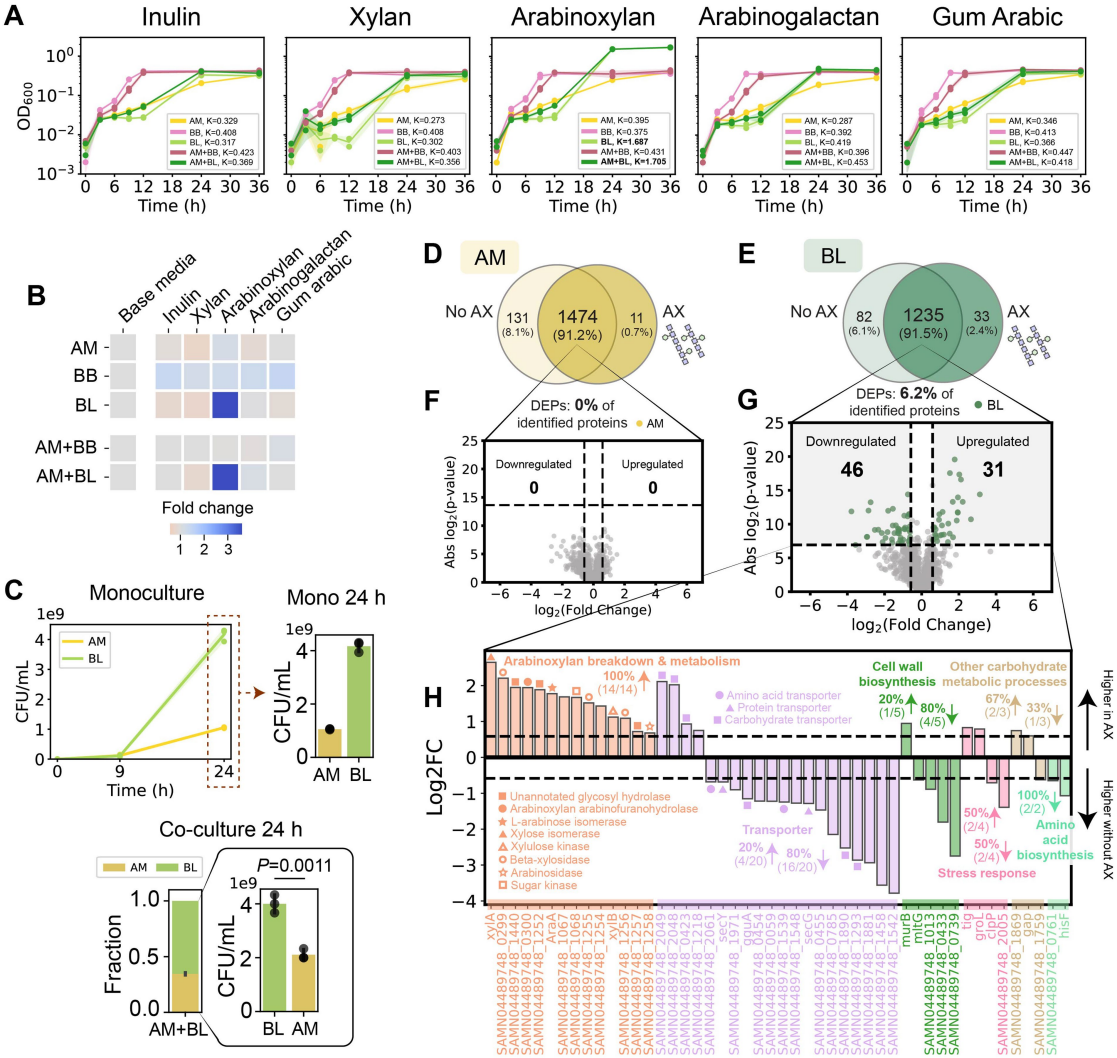
*Bifidobacterium longum* displays increased abundance and promotes the growth of *A. muciniphila* in the presence of arabinoxylan. **(A)** OD_600_ of AM, BB, BL, AM + BB, and AM + BL cultured in different dietary fibers measured over 36 h. Individual data points were shown. Lines represent the mean, and shading represents s.d. The maximum carrying capacity (K) of each species in the respective media is shown in the figure panels. **(B)** Heatmap of fold change of the Area Under the Curve (AUC) for each monoculture and co-culture when cultured in the presence of dietary fibers compared to growth in the base media alone. AUC data were extracted from panel A and Supplementary Figure S6. **(C)** Absolute abundance of AM and BL monocultures (top), and stacked bar plot of relative abundance and bar plot of absolute abundance of AM and BL in co-culture (bottom) in the base media supplemented with arabinoxylan (AX) as measured by CFU counting. Each bar represents the average relative or absolute abundance of each species, and the error bars represent s.d. (*n* = 3). *p*-value from a two-sided unpaired Student *t*-test of absolute abundance between AM and BL is shown. **(D,E)** Venn diagram for proteome comparison of AM **(D)** and BL **(E)** in the presence vs. absence of AX. Proteins shown are detected in two out of three biological replicates. **(F,G)** Volcano plot for AM **(F)** and BL **(G)** in the presence vs. absence of AX. Benjamini–Hochberg (BH) multiple testing correction was applied to the *p*-values from a two-sided unpaired Student *t*-test to control the false discovery rate (FDR) at 10%. In addition to the BH-corrected *p*-value constraint, differentially expressed proteins (DEPs) are defined to be those with an absolute fold change greater than 1.5, corresponding to the colored dots. The list of DEPs is shown in Supplementary Table S15. H, Log-transformed fold changes of selected DEPs in BL that were expressed higher in the presence of AX (positive values) or in the absence of AX (negative values). Vertical dashed lines indicate a 1.5-fold change.

We hypothesized that providing resources accessible only to BL would alleviate the reduced growth of BL in co-culture with AM compared to monoculture (Figures 1D–G). To this end, we screened for health-relevant diet-derived glycans that can be exclusively utilized by BL (Figures 4A,B; Supplementary Figure S6). We used inulin, xylan, arabinoxylan, arabinogalactan, and gum Arabic. Inulin (found in chicory root, artichoke, bananas, and onions) and acacia gum (arabinogalactan is a major component) have been demonstrated to increase both butyrate production in human subjects and health-relevant *Bifidobacteria in vitro* (Phillips et al., 2008; Kaddam and Kaddam, 2020; Belenguer et al., 2006). Xylan, an abundant component of cereal grains, has been shown to exhibit health-promoting properties (Smith and Melrose, 2022). Arabinoxylan (AX) is an important constituent of hemicelluloses in the endosperm and outer layers of cereal grains, including corn, wheat, rye, barley, oat, and rice. Of the five tested glycans, BL displayed a high degree of growth in the presence of AX (Figures 4A,B; Supplementary Figure S6) and exhibited a higher relative abundance than AM in co-culture (Figure 4C). By contrast, AM and BB cannot utilize AX in monoculture. Notably, in the presence of AX, BL increased the abundance of AM after 24 h of growth in co-culture compared to monoculture, likely via cross-feeding of sugars liberated from AX (Figure 4C).

### *Bifidobacterium longum* increases the expression of *A. muciniphila*’s Amuc_1100 in the presence of arabinoxylan

**Figure 5:**
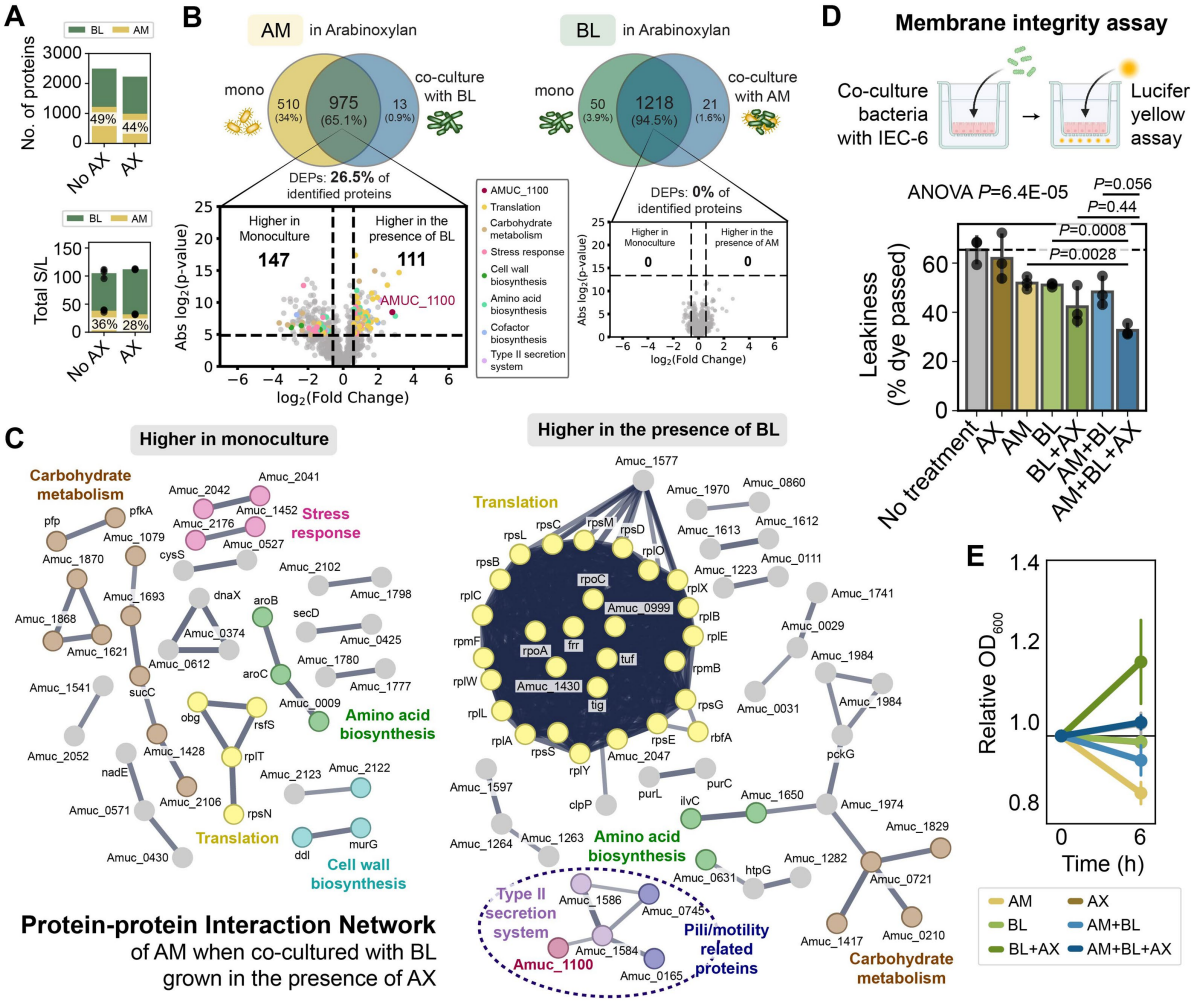
*Bifidobacterium longum* causes massive alterations in *A. muciniphila* protein expression in the presence of arabinoxylan, increases Amuc_1100 expression, and enhances the barrier integrity of intestinal epithelial cells. **(A)** Number of identified proteins and total normalized spectral counts of the whole proteins in the AM + BL co-culture in the presence and absence of AX. The percentage shows the fraction of AM’s protein identified in the co-culture (top) and the fraction of total normalized spectral counts of proteins belonging to AM in the co-culture (bottom). **(B)** Proteome comparison of AM or BL in monoculture vs. AM + BL co-culture in the presence of AX. The volcano plot shows proteins that are expressed higher in co-culture vs. monoculture, and proteins belonging to different biological processes are shown with different colors. The annotated protein (dark red) is Amuc_1100. The list of DEPs is shown in Supplementary Table S16. **(C)** Protein–protein interaction networks of AM that are expressed higher in monoculture than co-culture with BL (left) and higher in co-culture with BL than monoculture (right) when cultured in the presence of AX. Lines represent protein interactions (thicker indicates higher confidence), and colors represent protein functions. Nodes without a specific function are colored gray. Only high-confidence interactions are shown (STRING interaction score >0.7). **(D,E)** Bar plot of the leakiness of intestinal epithelial cells (calculated from the % of lucifer yellow dye that passed through the cells monolayer) after incubation with different bacterial samples **(D)** and the relative OD_600_ of the bacterial samples before and after incubation with the IEC-6 cells **(E)** (mean ± s.d., *n* = 3). One-way ANOVA followed by a post-hoc test using Bonferroni correction was used. Bonferroni-adjusted *p*-values from a two-sided unpaired Student *t*-test are shown.

While the proteome profile of AM was similar in the presence and absence of AX in monoculture (Figures 4D,F), BL up-regulated enzymes for AX degradation and arabinose/xylose utilization in the presence of AX (Figures 4E,G–H; Supplementary Table S15). In addition, we also observed some other changes in the biological processes of BL in the presence of AX (e.g., proteins for cell wall biosynthesis, stress response, metabolic processes, amino acid biosynthesis, and transporters), possibly associated with the cost of producing those enzymes (Figure 4H). In the presence of AX, BL displayed higher protein identification and total protein expression in AM + BL co-culture compared to the absence of AX (Figure 5A). Unlike in the Glc + GlcNAc or mucin media, AM did not alter the proteome of BL in the presence of AX (Figure 5B). On the other hand, BL led to substantial alteration in the proteome of AM (Figures 5B,C; Supplementary Table S16). Several proteins involved in carbohydrate metabolism, stress response, and cell wall biosynthesis were expressed at higher levels in AM monoculture, whereas proteins involved in translation and pili or type II secretion were expressed at higher levels when co-cultured with BL. These pili/type II secretion-related proteins were reported to be important for utilizing mucin (Davey et al., 2023), and thus, our results suggest that these proteins might be involved in other processes beyond mucin utilization. Notably, the membrane protein Amuc_1100 was up-regulated in the presence of BL (Figures 5B,C; Supplementary Table S16). Amuc_1100 is one of the most important proteins in AM that activates TLR-2 in IECs, improves gut barrier integrity, and was reported to replicate most of the beneficial effects of the bacteria (Plovier et al., 2017; Ottman et al., 2017).

### *Akkermansia muciniphila* and *B. longum* co-culture enhances the barrier integrity of intestinal epithelial cells in the presence of arabinoxylan

Previous studies have shown that AM (Reunanen et al., 2015) and BL (Srutkova et al., 2015) enhance the barrier integrity of IECs through different mechanisms. AM adheres to the intestinal lining, and its outer membrane protein Amuc_1100 binds to TLR2 in IECs to activate the downstream signaling pathway, including the expression of tight junction proteins. By contrast, BL enhances barrier integrity by modulating immune responses and secreting extracellular vesicles (Nie et al., 2025). To compare the membrane integrity-enhancing activity of AM, BL, and AM + BL in the presence and absence of AX, we co-cultured the bacteria with IEC-6 cells and performed a permeability assay using a previously established protocol (Gildea et al., 2017; Fan et al., 2021; Fan et al., 2020). Although Caco-2 cells (derived from human colon cancer) have been used as a cell model in the studies of intestinal barrier function (Wilson et al., 1990), the transepithelial electrical resistance (TEER) value of the Caco-2 cell monolayer (~900 Ω cm^2^) is much higher than that of the intestinal epithelium (~40 Ω cm^2^) (Shi and Zhao, 2019). Thus, the practicability of Caco-2 cells as a cellular permeability model might have some limitations. For this reason, IEC-6 cells [originated from the rat small intestinal crypt (Quaroni et al., 1979)] have been used to study the proliferation and differentiation of IECs since they better replicate the extracellular transport characteristics of intestinal epithelial cells (Fan et al., 2021). The TEER value of the IEC-6 cell monolayer is lower than that of Caco-2 but closer to the actual situation of the small intestine.

AM, BL, and AM + BL displayed similar positive effects on membrane integrity (~14 to 17% decrease in leakiness), whereas supplementing AX to the AM + BL pairwise community during incubation with IEC-6 cells increased the positive effect (~33% decrease in leakiness) (Figure 5D). Supplementing AX alone did not affect the membrane integrity, and the reduction in permeability due to AM + BL + AX treatment was significantly larger than AM or BL alone. This could be because AX enhanced the growth of BL when incubated with IEC-6 cells (Figure 5E), which subsequently promoted the growth of AM and up-regulated the expression of Amuc_1100 as observed from our proteomics data. In sum, our results show that the AM + BL pairwise community, with AX supplementation, led to the highest improvement in the barrier integrity of IECs.

To investigate the changes in protein expression of IEC-6 cells upon co-culture with AM, BL, and AM + BL in the presence and absence of AX, we subjected the IEC-6 cells that were treated with bacterial samples for 6 h to proteomics analysis (Supplementary Figure S7A). Treatment of IEC-6 cells with different bacterial samples led to a similar degree of proteome alterations (3.1, 4.5, 3.0, and 2.6% DEPs for IEC-6 cells treated with AM, BL, AM + BL, and AM + BL in AX compared to untreated cells, respectively) (Supplementary Figure S7B; Supplementary Tables S17–20). However, our proteomics approach was unable to detect most of the tight junction proteins (peptide spectrum match (PSM) value of 0), such as Claudins (Cldn-1, Cldn-18, Cldn-19, Cldn-3, Cldn-16, Cldn-5, Cldn-7, and Cldn-11) and Occludin, although we detected Cldn-15 in low abundance (PSM values of 1–2 across samples). This suggests that their expression levels were low under our experimental conditions, and/or enrichment steps during sample preparation are needed to increase their abundance in the samples. Junctional adhesion molecules (JAMs) were also detected in low abundance (average PSM of 2 across all samples for JAM-C or JAM-3, and average PSM of 3 for JAM-A or JAM-1, whereas JAM-4 was not detected with PSM of 0 across all samples). We also analyzed other junction proteins, including adheren junction proteins and desmosome-related proteins, and gap junction proteins (Supplementary Figure S7C). Both AM and BL treatments led to the up-regulation of protein kinase C iota type (Prkci) by 2.1 and 2.8-fold for co-culture with AM and BL, respectively. Notably, the expression of Cytohesins, in particular Cyth-2, was uniquely high in the AM + BL + AX treatment group (up-regulated by ~5.6-fold) and was not observed in other groups. Further mechanistic study on how BL improves the efficacy of AM in the presence of AX and how this leads to the enhancement of gut barrier integrity, combined with further *in vivo* validation experiments, could have important therapeutic implications.

## Discussion

The success of AM supplementation in treating multiple diseases in mouse models (Everard et al., 2013; Zhao et al., 2017; Wu et al., 2017; Depommier et al., 2020; Qu et al., 2021; Bian et al., 2019) has led to two clinical trials in overweight/obese insulin-resistant individuals (Depommier et al., 2019) and patients with overweight/obese type 2 diabetes (Zhang et al., 2025). However, in some cases, AM supplementation failed to modulate the dysbiotic gut microbiota composition in obese/diabetic rodents (Everard et al., 2013) and humans (Depommier et al., 2019; Zhang et al., 2025). Besides, some studies reported that certain beneficial effects of live AM on the host were lower compared to pasteurized AM (Depommier et al., 2019). Live AM is more attractive than pasteurized AM due to the beneficial secreted metabolites (e.g., SCFAs, P9 protein, extracellular vesicles) and more sustainable effects on host metabolism and health if stably engrafted. A study showed that the efficacy of AM supplementation in suppressing liver tumors in metabolic dysfunction-associated fatty liver disease (MAFLD)-hepatocellular carcinoma (HCC) mouse model was not optimal and can be enhanced by combining with PD1 therapy (Wu et al., 2025). Thus, the efficacy of AM could still be improved. The effects of introducing probiotic bacteria depend on the availability of ecological niches and the complex interactions with the resident gut bacteria. Single probiotic strains are not robust to environmental variability, such as variation in gut microbiome compositions, host genetics, immune responses, and diet, which could lead to variability in colonization and efficacy across individuals. Combining AM with other species that promote the colonization and abundance of AM in the gut, along with the production of beneficial proteins and metabolites, could improve its efficacy and effects on the gut microbiome.

Recently, interest has shifted from single-strain probiotics to the formulation of microbial consortia that are more robust to environmental variability and could provide orthogonal health benefits to the host (Sulaiman et al., 2024; Clark et al., 2021; Connors et al., 2025; Connors et al., 2023; Thompson et al., 2023). However, formulating stable consortia with desired specifications is challenging. In nutrient environments that contain mostly shared resources, the degree of resource competition is high among community members. Thus, certain strains, especially those with low growth rates, would be at a low abundance or even outcompeted by the community. Specific diets can be used to manipulate the abundance of the gut microbiota. For instance, polyphenols, alkaloids, capsaicin, plant-derived carbohydrates, and some Chinese Medicines have been shown to increase the abundance of AM (Yue et al., 2022). In this study, we showed that specific glycans can serve as a resource niche for species that are able to cleave the glycosidic linkages, and the subsequent degradation products will be available for other community members to consume. Thus, adding specific glycans is a promising strategy to increase the fraction of low-abundance species in a community by prioritizing them over the resources, and at the same time, benefiting other community members from the glycan-liberated sugars. From a therapeutic point of view, glycans can help circumvent the “priority effect” (Fukami, 2015), where resident communities exclude newly introduced species by occupying all available ecological niches of the newly introduced species. While defined communities with lower species richness may have difficulties colonizing the gut microbiome (Frese et al., 2012), glycans could serve as an orthogonal niche for the probiotic consortia and help them colonize the gut. Incorporating specific glycans that boost the community function can lead to the formulation of next-generation synbiotics (Swanson et al., 2020).

Previous studies have shown that AM and *Bifidobacterium* spp. are highly effective in improving host metabolism and are potential LBPs for treating metabolic diseases (Niu et al., 2024; Yan et al., 2021; Depommier et al., 2019; Zhang et al., 2025; Solito et al., 2021; Minami et al., 2015; Schellekens et al., 2021). Although there were attempts to combine these strains as a therapeutic community (Vedor et al., 2023; Nian et al., 2023), we lack an understanding of their interactions, growth, and stability in co-culture, and whether their functionality can be improved. This study elucidated the growth and proteome profiles of AM in co-culture with BB and BL in media containing different carbon sources (monosaccharides, mucin, or dietary fibers such as AX). While BB negatively impacted the growth of AM compared to monoculture and substantially affected its proteome profile in co-culture compared to monoculture when provided with monosaccharides, it was not the case when mucin was supplemented to the media. The proteome alteration of AM might be caused by resource competition with BB, which is better at utilizing the resources in the media with Glc + GlcNAc (Figures 1B,C). By contrast, mucin could serve as a niche for AM, hence partitioning the resources consumed by the two species and causing AM and BB to minimally affect each other’s proteome profile. Although BL is also better at utilizing Glc + GlcNAc compared to AM (Figures 1B,C), BL did not negatively impact AM’s growth nor significantly affect the proteome of AM. On the contrary, AM reduced the growth of BL in co-culture compared to monoculture (Figures 1D,E) and led to substantial alterations in the proteome of BL in the presence of Glc + GlcNAc (Figure 3G). This could be due to unknown toxic compounds or inhibitory metabolites, physical contact, or other mechanisms.

We demonstrated that providing the dietary fiber AX markedly enhanced BL’s abundance in the community. Notably, when supplemented with AX, BL promoted the growth of AM and increased the expression of the beneficial protein Amuc_1100, along with other proteins related to the type II secretion system and motility, leading to the enhancement of the barrier integrity of IECs. Our study serves as proof-of-concept that host and diet-derived glycans can specifically alter the growth and proteome profiles of major probiotic strains in co-cultures and can be harnessed for therapeutic applications. Further study could investigate the molecular mechanism governing the enhancement of growth and Amuc_1100 production of AM in the presence of BL and AX. In addition, we only used a simple system comprising AM and BB or BL in three different media containing monosaccharides, mucin, and AX. High-throughput approaches could be used to include more probiotic species or gut commensals, and more health-relevant dietary fibers or natural products to mine novel growth-promoting interactions that would be useful for designing next-generation therapeutic consortia.

## Methods

### Bacterial strain, media, and growth conditions

The strains used in this work were obtained from the sources listed in Supplementary Table S1. Two media formulations were tested to select the base media for this study: DeMan-Rogosa-Sharpe (MRS) broth (BD Difco™) and Brain Heart Infusion (BHI) broth (Thermo Scientific™ Oxoid™) (Derrien et al., 2004; Anand et al., 1984). BHI was chosen as the base media due to its ability to minimally support the growth of all species and allow growth enhancement when supplemented with specific additional carbon sources (Supplementary Figures S1A,B). For the experiments in this study, the base media was supplemented with either glucose (Glc) and N-acetyl-D-glucosamine (GlcNAc) as previously described (Plovier et al., 2017), 1% mucin, or 1% dietary fiber (inulin, xylan, arabinoxylan, arabinogalactan, or gum Arabic).

For all experiments, cells were cultured in an anaerobic chamber (Coy Lab products) with an atmosphere of 3.0 ± 0.5% H_2_, 15 ± 1% CO_2_, and balance N_2_ at 37 °C. Starter cultures were inoculated by adding 100 μL of a single-use 25% glycerol stock to 3 mL of BHI broth supplemented with Glc + GlcNAc, and cultured at 37 °C without shaking. All strains used in this study displayed an OD_600_ of ~0.8 or higher after overnight culture in this media condition (Supplementary Figure S1A).

### Cell cultures

Rat ileum epithelial IEC-6 cell line (CRL-1592) was purchased from the American Type Culture Collection (ATCC). The cells were cultured in Dulbecco’s modified Eagle’s medium (DMEM, 4.5 g/L glucose) (Gibco, Thermo Fisher Scientific) supplemented with 3.7 g/L NaHCO_3_, 10% (v/v) fetal bovine serum (FBS) (Gibco, Thermo Fisher Scientific), and 1% (v/v) Antibiotic-Antimycotic cocktail (penicillin, streptomycin, and Gibco Amphotericin B) (Gibco, Thermo Fisher Scientific). The cells were kept at 37 °C in 95% air/5% CO_2_ in a humidified incubator.

### Growth characterization in media with different carbon sources

Starter cultures of AM, BB, and BL were prepared. The cell pellets from starter cultures were collected by centrifugation at 3,000×*g* for 10 min, and then washed with the base media. The washed cell pellets were resuspended in the base media to a final OD_600_ of approximately 1. These cultures were inoculated into individual culture tubes containing the base media supplemented with specific carbon sources (Glc + GlcNAc, mucin, or dietary fiber) to an initial OD_600_ of 0.02. For monocultures, 60 μL of washed cultures was inoculated into 3 mL fresh media (50× dilution). For co-cultures, AM and either BB or BL were inoculated to an equal ratio (OD_600_ of 0.01 each) by inoculating 30 μL of each strain into 3 mL fresh media. The cultures were incubated at 37 °C anaerobically, and cell growth was determined by monitoring the OD_600_ every 3 h using BioTek Synergy H1 multimode reader (Agilent).

### Absolute abundance determination by colony-forming unit (CFU) counting

Absolute abundance could be calculated using OD_600_ or CFU counting. While cellular traits such as cell adhesion, size, and shape can influence OD_600_ measurements (Stevenson et al., 2016), CFU counting is biased by cell adhesion, dormant sub-populations, growth selection on solid vs. liquid media, and growth stage (Jansson and Prosser, 1997; Volkmer and Heinemann, 2011; Ou et al., 2017). To complement the measurement of growth profile (OD_600_ using plate reader), we performed CFU counting to determine the absolute abundance of bacterial species. For pairwise communities, OD_600_ measurement only provides information on the total growth of the co-culture, whereas CFU counting could distinguish the abundance of the two species using selective plates.

BHI agar (Thermo Scientific™ Oxoid™) was used for plating and counting the total CFU of all species in monocultures and co-cultures (total absolute abundance). Since AM is unable to grow in MRS, MRS agar plates were used as *Bifidobacterium* selective plates to calculate the abundance of BB and BL in the AM + BB and AM + BL co-cultures, respectively. The abundance of AM in the co-cultures was calculated by subtracting the total CFU obtained from the BHI agar from the CFU of either BB or BL obtained from the MRS agar. Three technical replicates were performed for each biological replicate during plating and CFU counting.

### Sample preparation for proteomics

For proteomics analysis, monocultures and co-cultures of AM, BB, and BL were cultured anaerobically in the respective media for 24 h, since this growth period allows all species to reach maximum population size. For all proteomics experiments, three biological replicates were performed for each sample, including the control sample. The cell pellet was suspended in 300 μL of lysis buffer (8 M Urea, 50 mM Tris–HCl pH 8.0), frozen in liquid nitrogen, and sonicated for 5 min. The sample was centrifuged (16,000×*g* for 10 min) to remove cell debris and insoluble materials. An aliquot of the sample was taken for the BCA protein assay (Pierce™ BCA Protein Assay Kit). After protein quantification, the sample was reduced by dithiothreitol (DTT; 0.1 M final concentration) at 37 °C for 1 h. For shotgun proteomics, 150 μg of proteins were mixed with up to 250 μL of the exchange buffer (6 M Urea, 50 mM Tris–HCl pH 8.0, 600 mM guanidine HCl), transferred to an Amicon® filter device (Millipore, Darmstadt, Germany), and centrifuged (14,000×*g* for 20 min). The proteins in the filter device were alkylated with iodoacetamide (IAA, 50 mM in exchange buffer) in the dark for 20 min, and then centrifuged (14,000×*g* for 20 min). To reduce the urea concentration, 250 μL of 50 mM ammonium bicarbonate was added to the filter device and centrifuged (14,000×*g* for 20 min). This step was repeated once. Proteins were digested by sequencing-grade modified trypsin (1:50 w/w, Promega, Madison, WI) for 12 h at 37 °C. Then, the sample was acidified with 10% formic acid to a final concentration of 0.1% (v/v) and centrifuged at 16,000×*g* for 5 min. Finally, the samples were desalted by C18 reverse-phase ZipTip (Millipore, Darmstadt, Germany) and dried with SpeedVac (Eppendorf, Hamburg, Germany) for 30 min.

### Liquid chromatography

The samples were reconstituted in 25 μL water/acetonitrile/formic acid in a 97.9:2:0.1 ratio (v/v/v), and processed through Bruker nanoElute Ultra-High-Performance Liquid Chromatography (UHPLC; Bruker Daltonics, Bremen, Germany) coupled to a hybrid trapped ion mobility-quadrupole time-of-flight mass spectrometer (TimsTOF Pro, Bruker Daltonics, Bremen, Germany) via a nano-electrospray ion source (Captive Spray, Bruker Daltonics). A volume of 1 μL (approximately 200 ng of the protein digest) was injected into the UHPLC system and separated on an IonOpticks 25 cm Aurora Ultimate Series Emitter column with Captive Spray Insert (250 mm × 75 μm internal diameter, 120 Å pore size, 1.7 μm particle size C18) at a flow rate of 0.3 μL/min. The mobile phase composition is 0.1% formic acid in water for solvent A, and 0.1% formic acid in acetonitrile for solvent B. The gradient was applied from 2 to 5% of solvent B for 0.5 min, from 5 to 40% of solvent B for 26.5 min, and then from 40 to 95% of solvent B for 0.5 min. In the end, the mobile phase was kept at 95% of solvent B for 0.5 min, and then decreased to 2% of solvent B for 0.1 min. 2 min equilibration with 2% of solvent B was applied before the next injection.

### Mass spectrometry

A detailed description of the Bruker TimsTOF Pro mass spectrometer used in this work can be found in the literature (Meier et al., 2015; Meier et al., 2018). We set the accumulation and ramp time to 100 ms each and recorded mass spectra in the range from *m*/*z* 100 to 1,700 using the positive electrospray mode. The ion mobility was scanned from 0.85 to 1.30 Vs cm^−2^. The quadrupole isolation width was set to 2 Th for *m*/*z* < 700 and 3 Th for *m*/*z* > 700, and the collision energy was linearly increased from 27 to 45 eV as a function of increasing ion mobility. The overall acquisition cycle of 0.53 s comprised one full TIMS-MS scan and four Parallel Accumulation-Serial Fragmentation (PASEF) MS/MS scans. Low-abundance precursor ions with an intensity above a threshold of 2,500 counts but below a target value of 20,000 counts were repeatedly scheduled and otherwise dynamically excluded for 0.4 min. The TIMS dimension was calibrated linearly using three selected ions from the Agilent ESI LC/MS tuning mix (*m*/*z*, 1/K_0_: 622.0289, 0.9848 Vs cm^−2^; 922.0097, 1.1895 Vs cm^−2^; 1,221.9906, 1.3820 Vs cm^−2^) in positive mode.

### Sequence database searching of proteomics data

The raw data were converted to mgf files by Bruker Compass DataAnalysis (version 5.2), and subsequently converted to mzML files by msconvert of the ProteoWizard [version 3.0.23233 (Kessner et al., 2008)]. The mzML files were searched using Comet [version 2024.01 rev.0 (Eng et al., 2013)] against the *A. muciniphila* BAA-835, *B. breve* JCM1192, or *B. longum* JCM1217 protein sequence database obtained from UniProt (downloaded December 2024). For co-culture samples, the protein sequence of all strains in the co-culture was combined into a single database. The sequences of common contaminants, such as trypsin and human keratins, and decoy sequences generated by shuffling amino acid sequences between tryptic cleavage sites were added to the database. The decoy sequences in the database are used for the false discovery rate (FDR) estimation of the identified peptides. The search parameters criteria were set as follows: 40 ppm peptide mass tolerance, monoisotopic mass type, fully digested enzyme termini, 0.02 amu fragment bin tolerance, 0 amu fragment bin offset, carbamidomethylated cysteine, and oxidized methionine as the fixed and variable modifications, respectively. The search results from Comet were processed by PeptideProphet (Keller et al., 2002), iProphet, and ProteinProphet of the Trans-Proteomics Pipeline [TPP (Deutsch et al., 2010)] in the decoy-assisted non-parametric mode. Every mzML run was analyzed independently. Protein identifications were filtered at a false discovery rate of 0.01 as predicted by ProteinProphet. The mass spectrometry proteomics data have been deposited to ProteomeXchange via the PRIDE repository with the dataset identifier PXD060954 (monoculture and co-culture growth of AM, BB, and BL in the presence of different carbon sources) and PXD061633 (IEC-6 cells co-cultured with AM, BL, or AM + BL in the presence and absence of AX).

### Detecting and processing shared peptides between two species in the community

Some of the identified peptide sequences in pairwise co-culture samples could match the proteome database of both species (shared peptides), and the probability would be higher for short peptide sequences. It is not possible to determine which of the two species (or both) these shared peptides belong to. To know what peptide sequence could be shared between AM and BB or AM and BL in co-culture, we performed *in silico* tryptic digestion of the whole proteome of AM, BB, and BL using the Uniprot database (parameters: minimum residue count 5, max missed cleavage 2, minimum fragment mass 400 Da, maximum fragment mass 7,000 Da), and generated a list of all possible shared peptides between AM and BB or AM and BL using an in-house python script. The percentage of theoretically shared peptides across the proteome of AM and BB or AM and BL (0.222 and 0.218%, respectively) is much smaller than that of BB and BL (14.8%). Most of the shared peptides between AM and BB or BL are short peptides (~5 amino acids, which is the threshold we used in our Comet database searching) (Supplementary Figure S4).

We then searched whether the peptides identified in the co-culture proteomic samples contain any of the shared peptides. There is a very low number of shared peptides detected across our 21 proteomic datasets, ranging from 13 to 19 in each sample. To deal with the shared peptides, we took a conservative approach by assigning the spectra of that peptide to both species in the co-culture. The TPP algorithm will only consider peptides with enough evidence of sibling peptides identification and remove unconfident peptide assignments with a low number of sibling peptides.

### Label-free quantification of proteomics data by spectral counting

To analyze the proteomics data, we first compared the proteome profile of the samples between different carbon sources to observe how resource environments impact protein expression. Next, we compared the proteome profile of each species when cultured in monoculture versus co-culture under the same carbon source to reveal the effect of the partner species on protein expression of a particular strain.

The proteins identified in at least two out of three biological replicates were used for label-free quantification by spectral counting. Only proteins with average spectral counts (across all runs) of at least three were considered for quantification. The quantification of proteins was given by the normalized spectral abundance factor [NSAF (Paoletti et al., 2006)], where the number of peptide-spectrum matches (PSMs) for each protein divided by the length of the corresponding protein is normalized to the total number of PSMs divided by the length of protein for all identified proteins. Student’s *t*-test was employed on the NSAF values to detect differential expression between the two time points. Benjamini–Hochberg (BH) multiple testing correction was applied to the *t*-test *p* values to control the false-discovery rate (FDR) at 10% (Benjamini and Hochberg, 1995). To further reduce false discoveries and limit our attention to the more highly regulated proteins, only proteins with fold changes higher or lower than ±1.5-fold were considered differentially expressed in our subsequent analysis.

### Bioinformatics analysis

We visualize our proteomic data using principal component analysis (PCA) of the NSAF values using the PCA function from the sklearn package with centering and scaling in Python (Supplementary Figure S3). We added 95% confidence intervals by calculating correlation matrices for the three replicates of each sample and then adding these intervals to our plot using the matplotlib package in Python. To compare the protein expression profiles of a particular strain across different samples, we generated heatmaps of NSAF values of the proteins identified across all samples (Supplementary Figure S2). STRING version 12.0 (Szklarczyk et al., 2016) was used to predict the protein–protein interactions and to visualize the interactions among the differentially expressed proteins.

### Transepithelial permeability assay using lucifer yellow

IEC-6 cells were cultured in transwells with 0.4 μm pore size inserts (SPL Life Sciences) in DMEM medium until fully differentiated. The transepithelial permeability assay was conducted as previously reported (Gildea et al., 2017; Fan et al., 2021; Fan et al., 2020). The integrity of the cell monolayer was determined by measuring the TEER using a Millicell ERS-2 voltohmmeter (Merck Millipore) every 24 h. The medium was changed every 2 days until the TEER value reached ~50 Ω cm^2^. The cells in the inserts were washed twice with phosphate-buffered saline (PBS) to remove residual DMEM medium.

Co-culturing bacteria with IEC-6 cells was performed using a previously reported protocol (Reunanen et al., 2015). A previous study reported that there is no growth difference in AM under oxic and anaerobic conditions (Reunanen et al., 2015), whereas BL is a facultative anaerobe and thus could tolerate oxic conditions. Around 10^7^ CFU of AM, BL, or AM + BL (mixed in equal ratio of 5 × 10^6^ CFU each) were resuspended in either 1 mL of Hanks’ Balanced Salt Solution (HBSS) (Gibco, Thermo Fisher Scientific) or HBSS supplemented with 1% arabinoxylan. This bacterial seeding density is suitable for binding with the IEC-6 cells (Shi et al., 2022). The bacterial suspension was added to the apical side of the IEC-6 monolayer, whereas either HBSS or HBSS with arabinoxylan was added to the basolateral side. The IEC-6 cells and bacteria co-culture were incubated for 6 h at 37 °C in oxic conditions. Then, the bacterial suspension was removed from the IEC-6 cells, and the inserts were washed twice with fresh HBSS. For the lucifer yellow assay, 900 μL fresh HBSS was added to the basolateral side, and 200 μL of lucifer yellow dye (Sigma Aldrich) was added to the apical side of the IEC-6 cells and incubated for 2 h at 37 °C in oxic conditions. Transepithelial permeability was assessed by quantifying the % of dye that passed through the monolayer by fluorescence measurement of the basolateral side (485 nm excitation and 535 nm emission) and comparing the values with lucifer yellow standard with known concentration values.

## Data Availability

The mass spectrometry proteomics data have been deposited to ProteomeXchange via the PRIDE repository with the dataset identifier PXD060954 (monoculture and co-culture growth of AM, BB, and BL in the presence of different carbon sources) and PXD061633 (IEC-6 cells co-cultured with AM, BL, or AM + BL in the presence and absence of AX).
