# Supplementary material for: Proteomic study of Akkermansia muciniphila and Bifidobacterium species co-culture under different carbon sources
Source: Front Microbiol. 2025 Nov 6;16:1666747. doi: 10.3389/fmicb.2025.1666747 (PMC12631272; doi:10.3389/fmicb.2025.1666747)
Supplement: Supplementary file 1 [file Supplementary_file_1.pdf]

Supplementary Figures for

Proteomic study of *Akkermansia muciniphila* and *Bifidobacterium* species co-culture under different carbon sources

Jordy Evan Sulaiman [1, 2, *], Yuewei Zhan [3], Shuchen Wang [3], Ka Lun Lai [3], James Ho Wa Li [3], Daniel Ye Yutong [4, 5], Karl Wah Keung Tsim [4, 5], Kenneth King Yip Cheng [1, 2, *], Yong Lai [3, *], Henry Lam [3, *]

[1] Department of Health Technology and Informatics, The Hong Kong Polytechnic University, Hung Hom, Kowloon, Hong Kong

[2] Research Institute for Future Food (RiFood), The Hong Kong Polytechnic University, Hung Hom, Kowloon, Hong Kong

[3] Department of Chemical and Biological Engineering, The Hong Kong University of Science and Technology, Clear Water Bay, Hong Kong

[4] Center for Chinese Medicine, Division of Life Science, The Hong Kong University of Science and Technology, Clear Water Bay, Hong Kong

[5] State Key Laboratory of Molecular Neuroscience, Division of Life Science, The Hong Kong University of Science and Technology, Clear Water Bay, Hong Kong

[*] To whom correspondence should be addressed:

Jordy Evan Sulaiman (jordy-evan.sulaiman@polyu.edu.hk),

Kenneth King Yip Cheng (kenneth.ky.cheng@polyu.edu.hk),

Yong Lai (yonglai@ust.hk),

Henry Lam (kehlam@ust.hk)

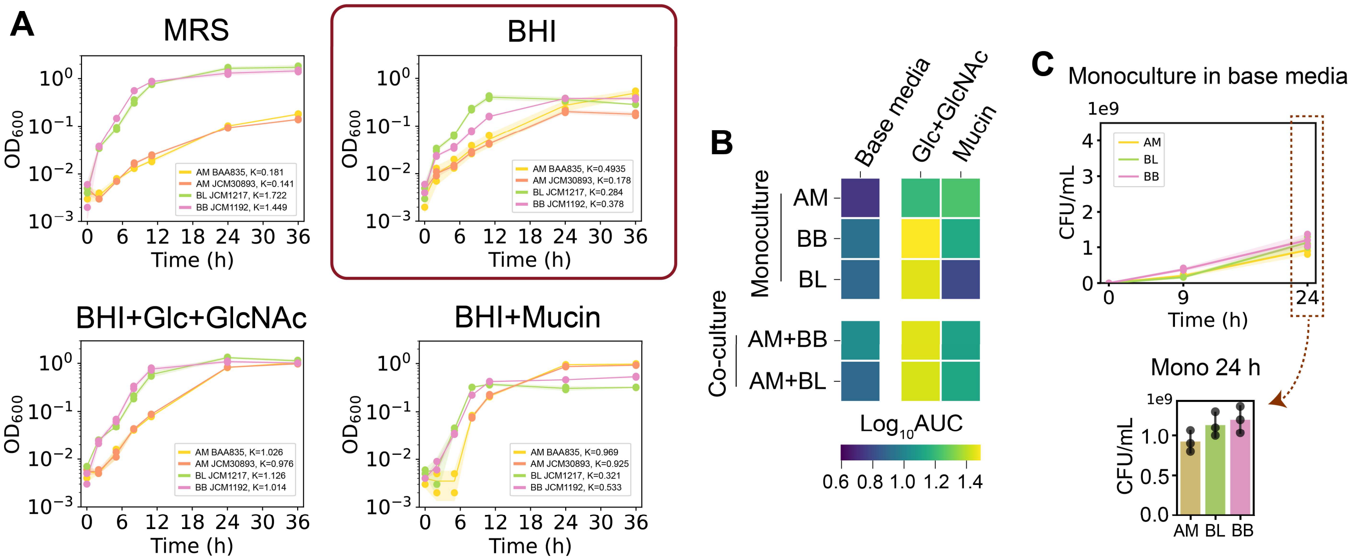

Supplementary Figure 1. Growth of monocultures and pairwise co-cultures in the presence of different carbon sources. A, Absolute abundance (OD_{600}) of two *A. muciniphila* (AM) strains (type strain BAA-835 and non-type strain JCM30893), *B. breve* (BB) JCM1192, and *B. longum* (BL) JCM1217, cultured in different media over 36 h. Individual data points were shown (n = 2). Lines represent the mean, and shading represents the standard deviation (s.d.). BHI was chosen as the base media (outlined with a red box). The maximum carrying capacity (K) of each species in the respective media is shown in the figure panels. **B**, Heatmap of the average integral OD_{600} or the Area Under the Curve (AUC) for each monoculture and co-culture extracted from growth data in the base media and base media supplemented with either Glc+GlcNAc or mucin after 36 h of growth from **Fig. 1B**. **C**, Absolute abundance of AM, BB, and BL monocultures in base media as measured by CFU counting (n=3).

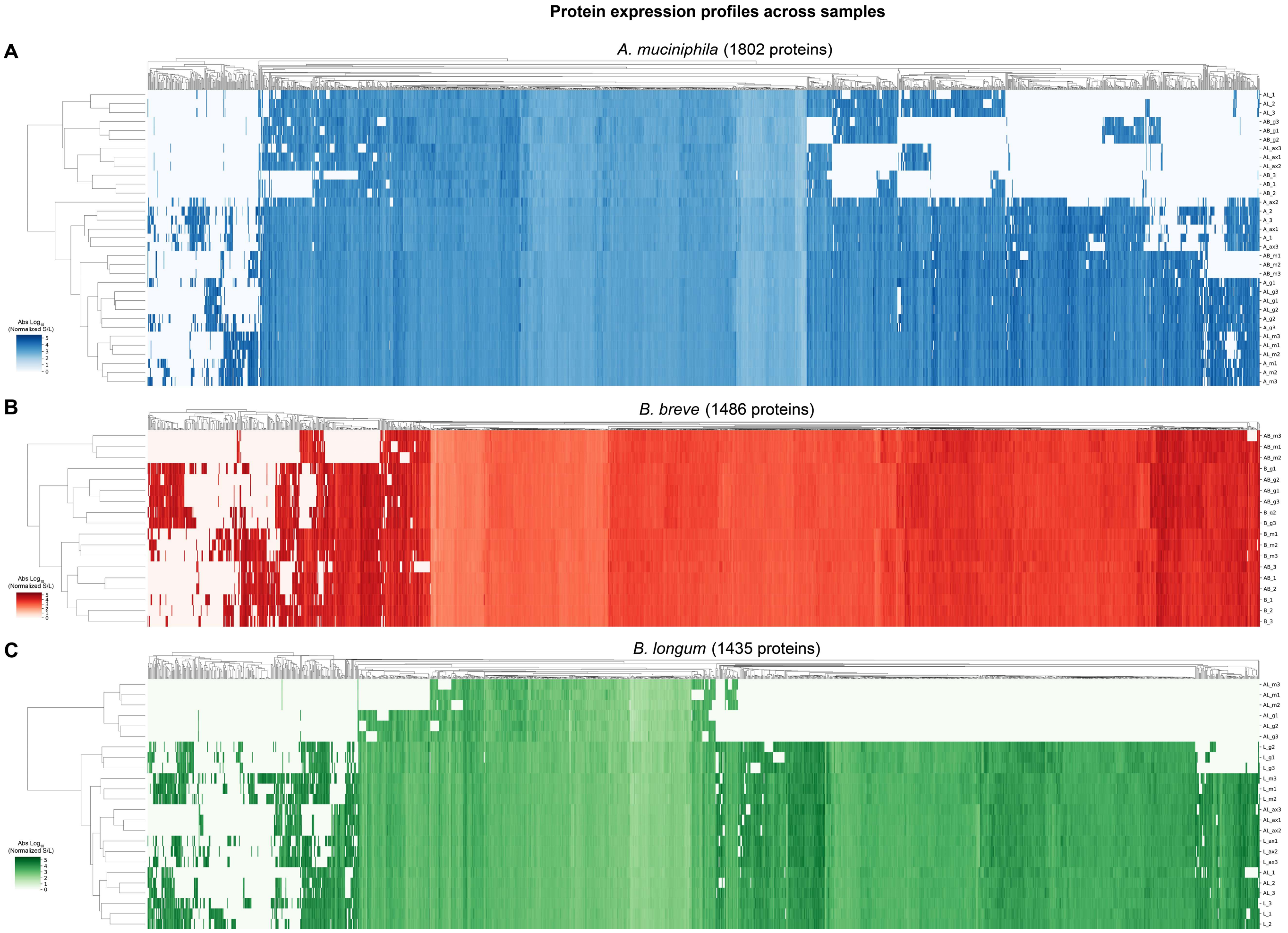

Supplementary Figure 2. Protein expression profiles across all *A. muciniphila, B. breve*, and *B. longum* samples. A-C, Biclustering heatmap of the log-transformed normalized spectral counts of proteins identified in AM (**A**), BB (**B**), and BL (**C**) across all samples in this study (monocultures and co-cultures in the presence of different carbon sources).

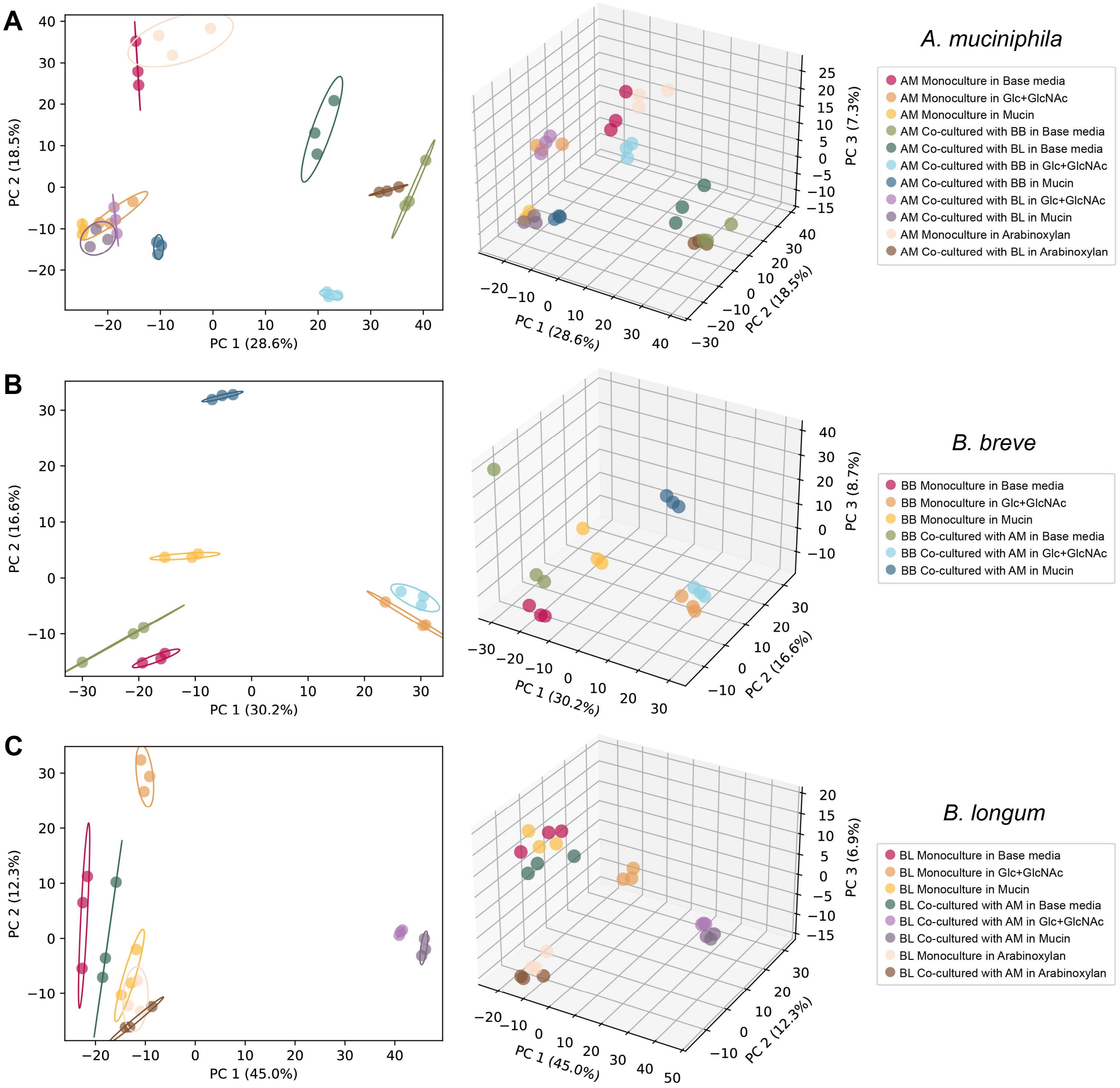

Supplementary Figure 3. Principal Component Analysis (PCA) of protein expression of *A. muciniphila, B. breve*, and *B. longum* across all samples. A-C, Projections of PC1 versus PC2 (left) and a three-dimensional projection of PC1, PC2, and PC3 (right) for AM (**A**), BB (**B**), and BL (**C**) are shown. Shaded circles represent 95% confidence intervals based on correlation matrices of the three biological replicates of each sample.

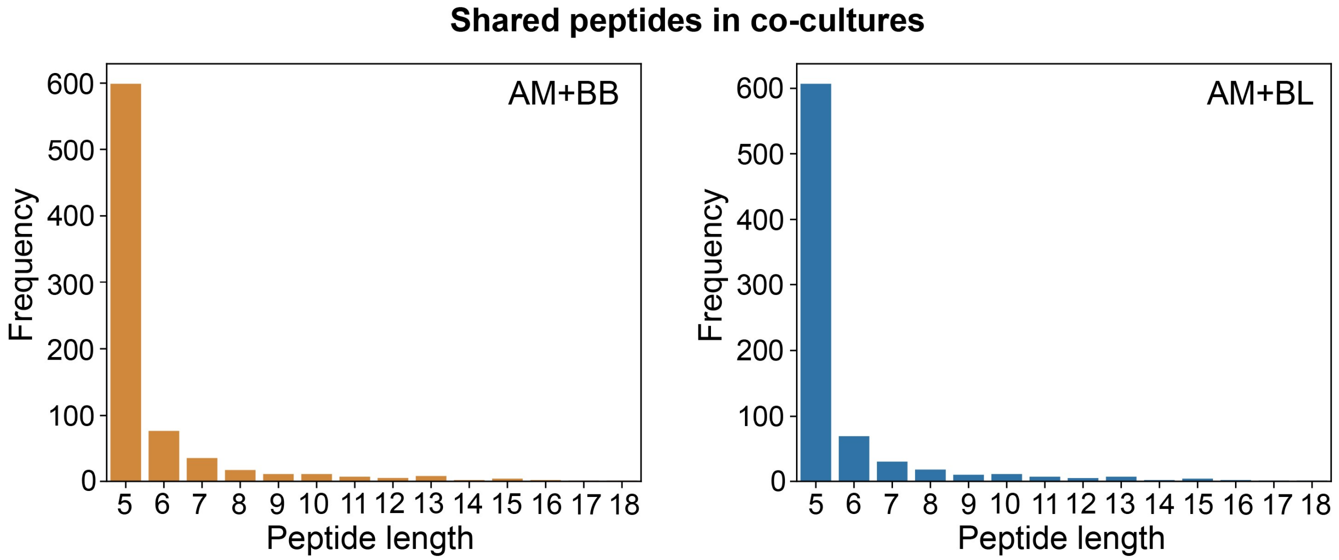

Supplementary Figure 4. *In silico* prediction of all shared peptides between two species in co-cultures. All possible shared peptides between AM and BB in AM+BB co-culture (left) or between AM and BL in AM+BL co-culture (right). The x-axis shows the length of the shared peptides, and the y-axis indicates the frequency of the shared peptides.

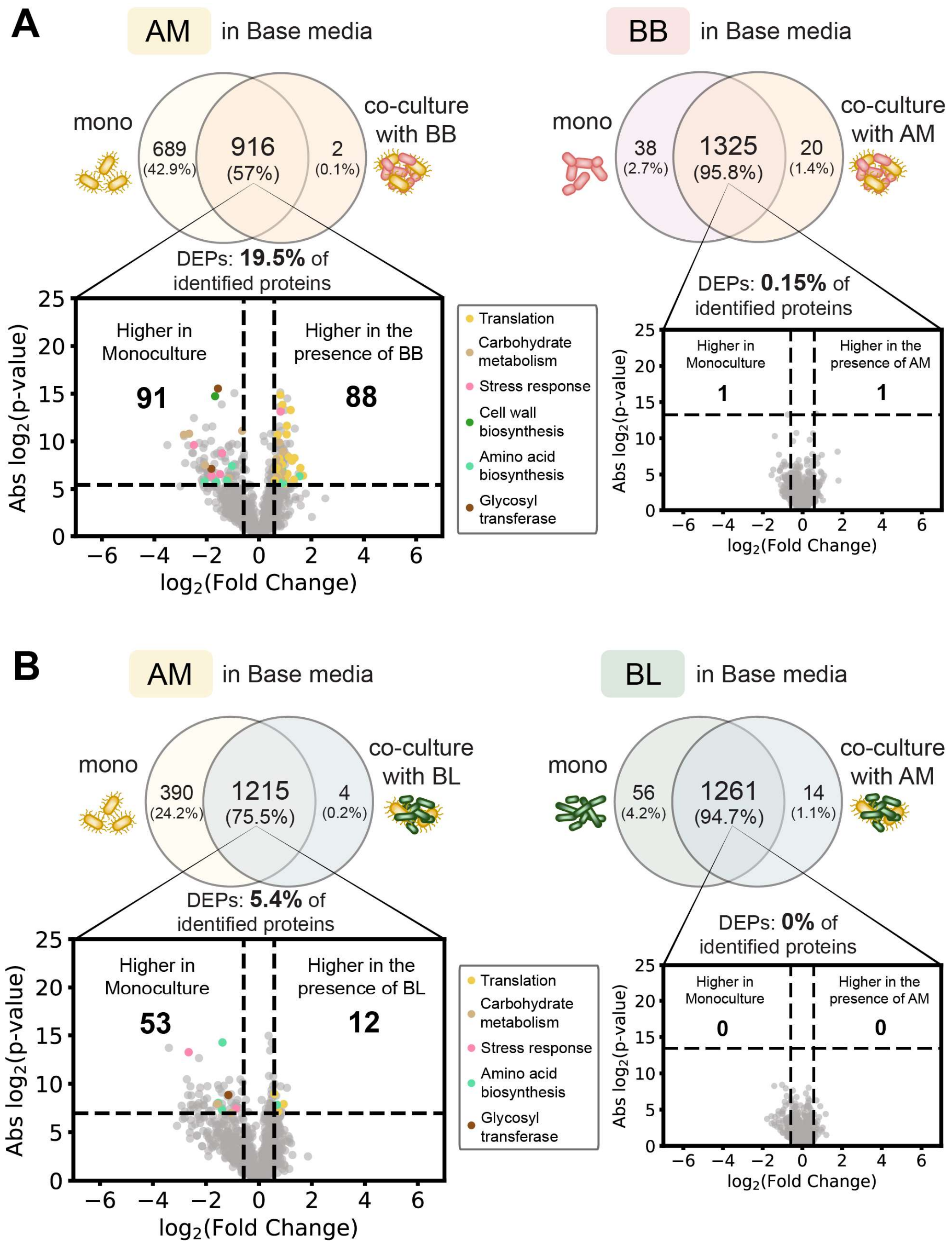

Supplementary Figure 5. Proteome comparison in co-culture versus monoculture in the base media. A-B, Proteome comparison of AM or BB in monoculture vs AM+BB co-culture in the base media (**A**), and AM or BL in monoculture vs AM+BL co-culture in the base media (**B**). The volcano plot shows proteins that are expressed higher in co-culture vs monoculture, and proteins belonging to different biological processes are shown with different colors. The list of DEPs is shown in **Table S12, S13**, and **S14**.

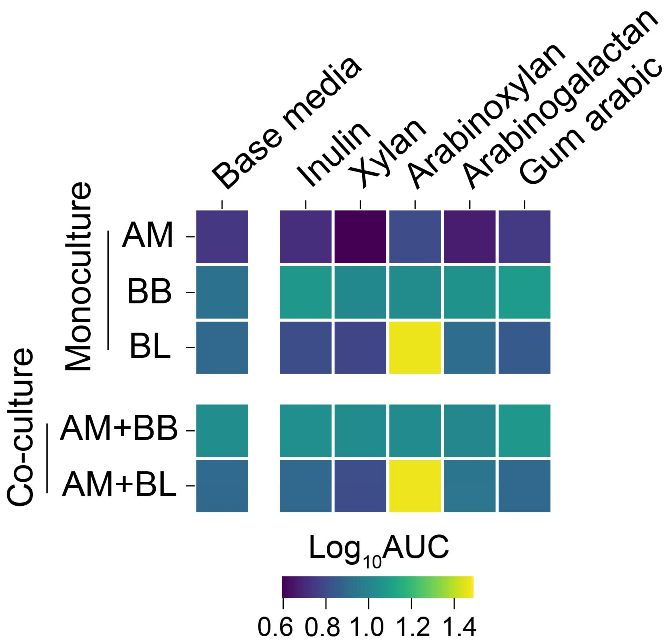

Supplementary Figure 6. Growth of AM, BB, and BL in base media supplemented with different dietary fibers. Heatmap of the average integral OD_{600} or the Area Under the Curve (AUC) for each monoculture and co-culture extracted from growth data in the base media and base media supplemented with different dietary fibers after 36 h of growth from **Fig. 4A**.

.

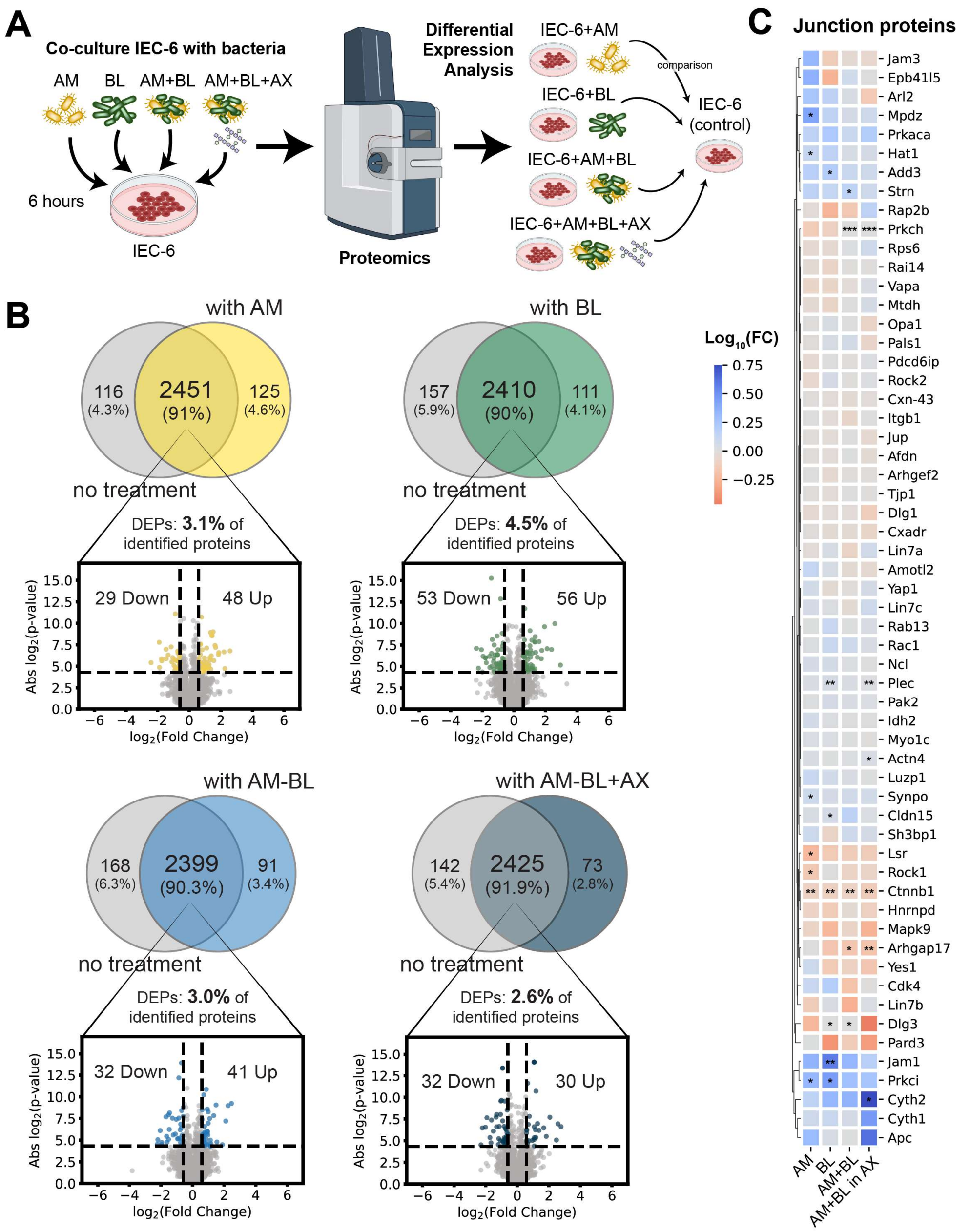

Supplementary Figure 7. Proteome profiling of IEC-6 cells in the presence of *A. muciniphila, B. longum,* or *A. muciniphila* and *B. longum* combination in the presence and absence of arabinoxylan (AX). A, Schematic of the proteomics experiment workflow on IEC-6 cells co-cultured with different bacterial samples for 6 h. **B**, Venn diagram for proteome comparison of IEC-6 cells after co-culturing with AM, BL, AM+BL, or AM+BL in the presence of AX for 6 h compared to untreated IEC-6 cells. Proteins shown are detected in two out of three biological replicates. The volcano plots show fold changes and *p*-values of IEC-6 proteins after co-culturing with AM, BL, AM+BL, or AM+BL in the presence of AX for 6 h compared to untreated IEC-6 cells. Differentially expressed proteins (DEPs) are defined to be those with an absolute fold change greater than 1.5 and Student *t*-test *p*-value less than 0.05, corresponding to the colored dots. The list of DEPs is shown in **Table S17** to **S20**. **C**, Heatmap of fold change of the junction proteins in IEC-6 cells after co-culturing with AM, BL, AM+BL, or AM+BL in the presence of AX for 6 h compared to untreated IEC-6 cells. Asterisks indicate statistical significance (Student *t*-test *p*-values): *, <0.05; **, <0.01; ***, <0.001.
